# The Role of Eye Gaze in Regulating Turn Taking in Conversations: A Systematized Review of Methods and Findings

**DOI:** 10.3389/fpsyg.2021.616471

**Published:** 2021-04-07

**Authors:** Ziedune Degutyte, Arlene Astell

**Affiliations:** Samsung AI Center, Cambridge, United Kingdom

**Keywords:** eye gaze, turn taking, dyads, triads, communication, conversation

## Abstract

Eye gaze plays an important role in communication but understanding of its actual function or functions and the methods used to elucidate this have varied considerably. This systematized review was undertaken to summarize both the proposed functions of eye gaze in conversations of healthy adults and the methodological approaches employed. The eligibility criteria were restricted to a healthy adult population and excluded studies that manipulated eye gaze behavior. A total of 29 articles—quantitative, qualitative and mixed methods were returned, with a wide range of methodological designs. The main areas of variability related to number of conversants, their familiarity and status, conversation topic, data collection tools—video and eye tracking—and definitions of eye gaze. The findings confirm that eye gaze facilitates turn yielding, plays a role in speech monitoring, prevents and repairs conversation breakdowns and facilitates intentional and unintentional speech interruptions. These findings were remarkably consistent given the variability in methods across the 29 articles. However, in relation to turn initiation, the results were less consistent, requiring further investigation. This review provides a starting point for future studies to make informed decisions about study methods for examining eye gaze and selecting variables of interest.

## Introduction

Human beings have evolved complex social-cognitive skills which enable us to exchange knowledge and communicate in multiple ways (Herrmann et al., 2007). People exchange verbal, vocal [e.g., tone of voice; (Lerner, 2004)] and non-verbal [e.g., eye gaze, gestures, facial expressions (Kendon, 1967; Bavelas and Chovil, 2000)] behaviors that convey meanings, intentions, and information. Non-verbal behavior can enrich conversation by adding extra information, or revealing emotional states that are not expressed verbally (Choi et al., 2005). Eye gaze in particular has been identified as playing a key role in communication, with infants showing a preference for direct gaze from birth (Farroni et al., 2002). The role that eye gaze plays in social interaction has been studied across a variety of fields, including typical and atypical child development (Baron-Cohen, 1997; Morales et al., 2000), mental health conditions [including schizophrenia (Dowiasch et al., 2016); posttraumatic stress disorder (Lazarov et al., 2019), and bipolar disorder (Purcell et al., 2018)], primates (Ryan et al., 2019) and human-robot interaction (Admoni and Scassellati, 2017). Additionally, eye gaze has been studied with different theoretical and methodological approaches from neuroscience (Sato et al., 2016) to sociology (McCarthy et al., 2008), producing a rich variety of data but complicating the conclusions that can be drawn about the role of eye-gaze in conversation.

Pioneering research conducted by Kendon (1967) suggested that eye gaze is used to regulate and monitor turn taking. Specifically, Kendon proposed that speakers tend to avert their gaze at the start of their turn in order to concentrate and plan their speech or to indicate that they are now holding the floor. He further proposed that in a two-person conversation, at the end of their turn the speaker gazes at the listener to indicate the end of their turn and to seek information about the listener's availability to speak next (Kendon, 1967).

A decade later Kendon's research was challenged in studies by Rutter et al. (1978) and Beattie (1978, 1979). Whilst Rutter et al. (1978) also found that at the end of the turns, speakers tended to gaze at the listener in a dyadic situation, they argued that in order to claim that eye gaze has a role in turn taking, the gaze pattern should follow three rules. Firstly, speakers should be looking at their conversation partners more at the end of their turns than at the beginning, because at the start of the turn the speakers should be gazing away to concentrate and plan their speech (Rutter et al., 1978). Secondly, at the end of one speaker's turn, the conversation partners should share a high level of mutual gaze, because in order for a speaker to pass the turn the listener should be available to receive it (Rutter et al., 1978). Finally, there should be higher levels of mutual gaze between conversation partners at the end of the turns rather than at the start of the new turns, because a new speaker at the start of their turn, should start gazing away to concentrate (Rutter et al., 1978). To test these predictions, Rutter et al. (1978) carried out two studies of which the first failed to support these three rules, and the second provided only partial support.

However, Rutter et al.'s (1978) approach to data analysis differed from Kendon's (1967) making direct comparison difficult. For example, to test the first rule—that the speakers should be looking at their conversation partners more at the end of their turns than at the beginning (because at the start of the turn the speakers should be gazing away to concentrate and plan their speech)—Rutter et al. (1978) compared the number of turns when the speaker was looking at the listener at the start of new utterance with the number of turns that the speaker was looking at the listener at the end of old utterance. In comparison, Kendon (1967) in attempting to identify if the speaker was looking at their conversation partners more at the end of their turns, compared the number of turns in which the speaker gazed toward the listener with the number of turns in which the speaker did not gaze toward the listener at the end of the turns.

Kendon (1978) findings were further challenged by Beattie (1978, 1979) in respect of proposed methodological limitations. Beattie (1978) pointed out that Kendon (1967) failed to provide the definition of “utterance” he used or the different types of gaze (i.e., prolonged, sustained). Furthermore, Beattie (1978) noted that in one of Kendon's (1967) analyses in which he was examining utterances with delayed responses, Kendon used data from only two out of the seven dyads and also reported data about “long utterances,” which actually included data of “all utterances.” Beattie's (1978) overall findings did not support Kendon's (1967) claim that eye gaze facilitates turn taking. In fact, Beattie (1978) found an opposite effect that more turns ended with gaze aversion.

Kendon (1978) responded by highlighting multiple differences in methodologies in the studies of Beattie (1978) and Rutter et al.'s (1978) that may have contributed to different findings between the studies. For example, both topic and type of conversation, differed between Rutter's first experiment and Kendon's study. Rutter et al. (1978) used 3-min segments from the beginning, middle and the end of a “getting acquainted” conversation. Kendon also used segments from a “getting acquainted” conversation but mainly concentrated on the segments toward the end of a 30 min conversation (Kendon, 1978). Kendon (1978) argued that Rutter et al.'s (1978) choice to include the first 3 min of conversation when people spent time exchanging details about themselves, may have affected their eye gaze behavior. For example, Exline et al. (1965) observed that when participants are asked very personal questions about their fears and desires, they are more likely to avoid mutual gaze than during non-personal ones. Kendon (1978) also noted differences in the status of the speakers between his study and Beattie's (1978), where the speakers were of unequal status, specifically a student and a supervisor. More recent investigation suggests that social status of one conversation partner affects eye gaze behavior of the other conversation partner, such that people with a high status tend to be observed more often and for a longer periods of time, than people with a lower status (Foulsham et al., 2010).

These three early studies (Kendon, 1967; Beattie, 1978; Rutter et al., 1978) highlighted the importance of study variables when designing studies of the role of eye gaze in conversation. Many further studies have also identified various factors that affect eye gaze direction during conversation. For example, the amount of gaze and direction tend to be affected by acquaintance status (Strongman and Champness, 1968; Rubin, 1970; Bissonnette, 1993), spatial arrangements between conversation partners (Argyle and Dean, 1965; Argyle et al., 1973; Blythe et al., 2018), gender (Argyle and Dean, 1965; Myszka, 1975; Bissonnette, 1993), cultural and ethnic factors (McCarthy et al., 2006; Rossano et al., 2009), conversation topic (Exline et al., 1965; Glenberg et al., 1998), and when experiencing different emotions (Kendon, 1967; Adams et al., 2005; Kleinke, 1986).

Understanding the role of eye gaze in turn-taking requires an understanding of how turns work in conversations. From a linguistic perspective turn-taking consists of many components and rules (for a full review see: Duncan, 1972; Sacks et al., 1974). Duncan (1972), proposed that turn taking is communicated through a set of rules and behavioral signals that both speakers and listeners follow. For example, the next speaker can take their speaking turn if the current speaker shows one or multiple “turn-yielding” signals. The turn-yielding signals include rising or falling intonation at the end of phonemic clause, a stressed syllable at the end of phonemic clause, turning the head toward the listener or/and stopping using hand gestures. However, if the current speaker wishes to continue and hold their turn, despite displaying some turn-yielding signals, the attempt-suppressing signal (i.e., turn holding) that consists of the speaker using hand gestures, almost always wins over (Duncan, 1972).

Conversation Analysis (CA; Sacks et al., 1974) was pioneered to study social interactions taking human actions and social context into consideration (for full review see: Goodwin and Heritage, 1990). Sacks et al. (1974), considered that turn taking is influenced by two components. The first, “Turn Constructional Unit” (TCU), defines a turn as a construct made of either sentential, clausal, lexical, or phrasal units. The speaker is permitted to finish one of these unit turns, and the first possible completion of this unit represents a “Transition Relevance Place” (TRP), where the next speaker may *take over* a speaking turn (Sacks et al., 1974). The second component is the “Turn Allocation” component, a technique used to *allocate* the next speaker. Sacks et al. (1974) proposed that a turn can be allocated either by the current speaker selecting the next speaker by using some sort of reference such as direct eye gaze or by listeners self-selecting themselves to be the next speaker. Furthermore, these two components are accompanied by rules similar to those described by Duncan (1972), such as if a current speaker selects the next speaker at the TRP, then the observing participants, i.e., those not selected by the current speaker to take the next turn, should not proceed. During self-selection the first speaker to speak would be granted a turn (Sacks et al., 1974).

Additionally, Schegloff and Sacks (1973), Sacks et al. (1974) and Schegloff (1972) proposed that types of action sequences play a role in next-speaker selection. These sequences consist of two parts that are relevant to each other, where the first part produced by one speaker, selects the next speaker to contribute to the second part (Schegloff and Sacks, 1973). These may include, question-answer sequence (Schegloff, 1972), greeting-greeting sequence (Schegloff, 1968), other-initiated repair sequences (Schegloff, 1997), or sequence-initiating actions (Robinson and Bolden, 2010). For example, during sequence-initiating actions, one speaker may offer a favor to another person, who is then obliged to refuse or accept. In this case the two parts would be offer-refusal/acceptance (Robinson and Bolden, 2010). Alternatively, during greeting-greeting sequences, one person may greet another person, which would oblige the other person to greet them back (Schegloff, 1968). Furthemore, work by Rossano (2012) revealed that participants' eye gaze behavior was most likely to be influenced by this sequential organization of the turns as proposed by Schegloff and Sacks (1973), Sacks et al. (1974), Schegloff (1972), and Schegloff (1997) and may operate in different ways when listening to simple questions than when listening to extended stories (Rossano, 2012). Referring back to the studies by Kendon (1967), Beattie (1978), and Rutter et al. (1978), they reported different definitions of turns and analyzed conversations consisting of variety of sentence types including greetings, questions and possible extended stories, which likely have influenced the findings.

Since the early days of eye gaze research many further studies have been undertaken using a wide range of methodological approaches to clarify and extend our understanding of eye gaze in conversation. Reflecting the importance of both methods *and* results in eye gaze research, the aim of this review is to (i) summarize findings of the role of eye gaze in relation to turn taking and (ii) to summarize the major methodological considerations in this field of research. The researchers hope that this review will benefit researchers new to this field, seeking to learn more about this subject or conduct their own research.

## Methods

A systematized review method was chosen because it aims to include elements of systematic review, such as comprehensive search, but has fewer restrictions for inclusion criteria (Grant and Booth, 2009). This suits the requirements of the current search to return the broad range of articles and methodologies employed in this field. A seven-step framework for systematic review was used for guidance, with four (research question, literature search, data extraction, results) out of the seven steps used in this review (Wright et al., 2007). The first step based on the framework of Wright et al. (2007) was to formulate a research question:

- Does the healthy adult population use eye gaze to regulate turn taking in conversations?

The second step was to conduct a literature search. A total of 20 search terms were created and combined to reflect the population of interest, exposure, and outcome (Table 1). To capture a broader range of literature appropriate Boolean search terms were used (Table 1). The literature search was carried out on two relevant databases in the psychology field: PsychINFO and Web of Science. As Web of Science consists of categories unrelated to psychology, to simplify the search, categories such as ophthalmology, neuroscience, engineering electrical electronic and zoology were excluded. The included Web of Science search categories were psychology experimental, psychology, psychology social, psychology biological, and psychology educational.

**Table 1:** A list of search terms.

**Population of interest “NOT”**	**Exposure “AND”**	**Outcome “AND”**
Infant^*^, child^*^, avatar^*^, virtual, robot^*^, disease^*^, disorder^*^	Dyads, dyadic, triads, triadic, group, turn taking, conversation, communicat^*^, interaction^*^	Eye gaze, gaz^*^, eye contact, eye pattern

The symbol "*" provides a variety of affixes to the stem of the key word that helps to expand the search in the databases.

**Figure 1:**
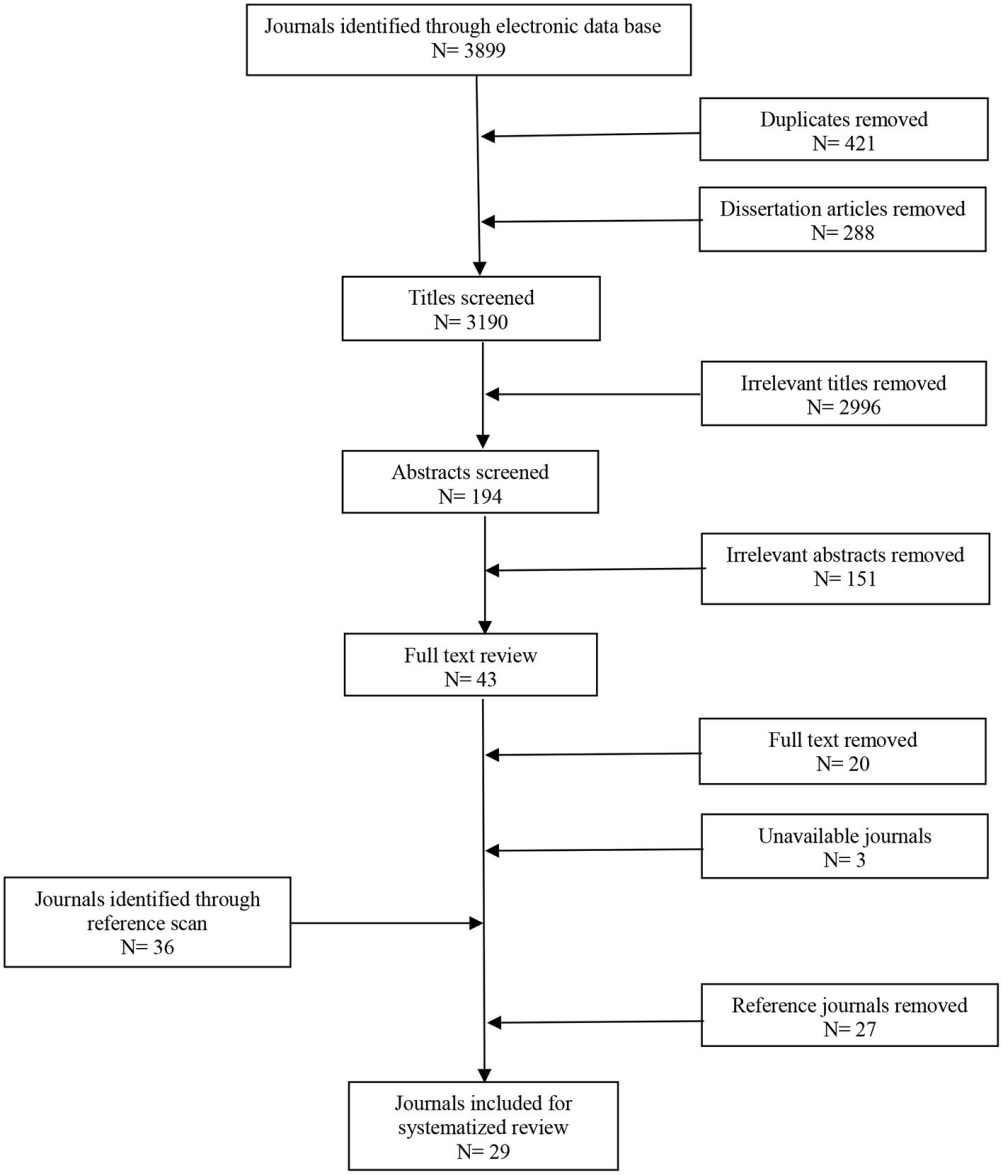
Flow diagram of search procedure.

No restriction was placed on the date of publication to cover the evolution of research into the role of eye gaze in turn taking. The included papers spanned qualitative, quantitative, and mixed methods. All included studies observed human to human conversations to measure gaze direction within speech turns. Studies that manipulated participants' gaze behavior (i.e., instructed to stare at the partner) and studies on animals, children, robots, and all mental health or cognitive disorders where excluded. The search was conducted in the last week of May 2019. The data extraction was performed on 3,899 retrieved papers. A total of 421 duplicates and 288 unpublished dissertation articles were removed. The remaining papers were journal articles, book chapters and conference journal papers. The review only included original research papers, therefore book chapters summarizing findings of other studies were excluded. After reviewing titles and abstracts, 3,147 papers were excluded due to not meeting criteria. A total of 43 papers were further investigated and after applying exclusion criteria, 20 papers were selected for final analysis. A further hand search was carried out by scanning the publication titles in the reference of the selected 20 papers and then reading abstracts of the selected titles. This process identified an extra nine papers for inclusion (Figure 1).

The third step was to conduct data extraction. Each of the 29 selected papers was read in full and information regarding research methods, such as language the study was conducted in, participant demographics and study procedures was recorded in a spreadsheet. Information was also recorded about the data collection method, definitions of eye gaze used, and coding schemes applied to the data. The researchers noted all the information about eye gaze patterns in relation to different features of turn taking (Table 2) which were then formed into groups. The final step based on the systematic review framework (Wright et al., 2007) was reporting the results.

**Table 2:** Key words and definitions.

**Key words**	**Definitions**
‘Turn Constructional Unit’ (TCU)	A turn construct made of either sentential, clausal, lexical, or phrasal units (Sacks et al., 1974).
‘Transition Relevance Place’ (TRP)	A transition place after TCU, where the next speaker may take over a speaking turn (Sacks et al., 1974).
Exchange sequences	Sequences are made of two parts that are relevant to each other, where the first part produced by one speaker, selects the next speaker to contribute to the second part (Schegloff and Sacks, 1973). These may include, question-answer sequence (Schegloff, 1972), greeting-greeting sequence Schegloff, 1968, other-initiated repair sequences (Schegloff, 1997), or sequence-initiating actions (Robinson and Bolden, 2010).
Turn start	The speaker starts talking.
Turn give, turn yield, floor switch, end of the turn	The speaker has finished talking and is letting the next speaker take a turn.
Turn hold, floor hold	The speaker continues to talk after TRP.
Interruption, overlap	Simultaneous speech between conversation partners.
Backchannels, feedback responses, accompaniment signals	The listener uses short verbal and non-verbal responses during speech to acknowledge the speaker (i.e., nods, short segments of speech “mhm,” “yeah.”)
Breakdowns	The speaker has to repeat a segment of speech, because a conversation partner misunderstood, was unable to hear or did not focus attention on the speaker.
Hesitant speech	Speech that is difficult to construct, consisting of brief pauses that is likely to be accompanied with verbal or vocal hesitation markers, such as “mhm.”
Switching pause	A pause between turns.
Mutual gaze, eye contact	Two conversation partners looking at each other at the same time.
Gaze shift, gaze transition	A movement of eyes toward or away, from a person or object.
Gaze ratio	An amount of time spent gazing toward or away, from a person or object.

## Results

### Study Characteristics

There was great variation between studies in the level of detail provided with multiple papers omitting important details about design and participants or the rationale for their methodological decisions. The studies varied in size from five to 69 participants (Table 3), but only 11 studies reported participant's ages (Lamb, 1981; Harrigan and Steffen, 1983; Harrigan, 1985; Egbert, 1996; Rossano et al., 2009; Eberhard and Nicholson, 2010; Cummins, 2012; Jokinen et al., 2013; Ho et al., 2015; Holler and Kendrick, 2015; Brône et al., 2017), which ranged between 18 and 65 years. Fourteen of the 29 studies examined conversation in dyads, 11 looked at triads, six studied multiparty conversations, and one did not report the number of interactants (Table 3). In 17 of the studies the participants were acquainted with each other, in eight the participants were unacquainted, and eight did not specify the relationship between participants (Table 3). Seven studies reported only same sex conversations, seven reported only mixed sex conversations, four studies reported both same and mixed sex conversations, and 12 studies did not specify (Table 3).

**Table 3:** Description of interactions.

	**Study setting**		**Number of participants**	**Number of interactants**			**Conversation type**				**Acquaintances**	
	**Laboratory**	**Ecological**		**Dyad**	**Triad**	**Multiparty**	**Female only**	**Male only**	**Mixed sex**	**Same sex**	**Acquainted**	**Unacquainted**
Kendon (1967)	o		13	o								o
Beattie (1978)	o		5	o				o			o	
Rutter et al. (1978) Study 1	o		36	o			o	o	o			
Rutter et al. (1978) Study 2	o		48	o			o	o			o	o
Beattie (1979)	o		5	o				o			o	
Goodwin (1980)		o				o						
Lamb (1981)	o		75	o	o					o		o
Harrigan and Steffen (1983)	o		5			o			o		o	
Harrigan (1985)	o		5			o			o		o	
Kalma (1992)	o		69		o		o	o				
Egbert (1996)		o	41			o					o	
Novick et al. (1996)	o		8	o			o	o				o
Bavelas et al. (2002)	o		18	o			o	o	o			o
Lerner (2003)		o				o					o	
Jokinen et al. (2009)	o		6		o			o	o		o	
Rossano et al. (2009)		o	55	o							o	
Eberhard and Nicholson (2010)	o		14	o			o	o			o	o
Cummins (2012)	o		11	o							o	
Jokinen et al. (2013)	o		6		o						o	
Streeck (2014)		o									o	o
Ho et al. (2015)	o		40	o								o
Holler and Kendrick (2015)	o		21		o		o	o	o		o	
Park (2015)		o	22	o								
Brône et al. (2017)	o		40	o	o				o		o	
Kendrick and Holler (2017)	o				o						o	
Auer (2018)	o		6		o						o	
Blythe et al. (2018)		o				o			o			
Ijuin et al. (2018)	o		60		o				o			
Weiss (2018)	o		24		o				o			
Zima et al. (2019)	o		30		o				o			

*The symbol “o” represents the feature was present*.

Seventeen of the 29 studies were quantitative, eight were qualitative, and four used a mixed methods design (Table 4). Fourteen studies using quantitative design reported reliability scores (ranging between 0.46 kappa and 100%) or provided information on how they assessed agreement between multiple coders to correct disagreements (Table 4). The remaining three quantitative studies did not report reliability results (Table 4). Two out of four mixed method studies reported how they looked for agreement between multiple coders to correct disagreements (Table 4). Reliability checks were not reported in the qualitative studies (Table 4), as is a common practice (McDonald et al., 2019).

**Table 4:** Characteristics of study designs and manipulations.

	**Design**	**Inter-rater** **reliability**	**Language**	**Conversation topic**			**Length of coded data**	**Eye tracking**
				**Free flowing**	**Specific topic**	**Other**		
Kendon (1967)	Quantitative		English university	o			5–9 min	
Beattie (1978)	Quantitative	98–100% on speech; 97% on gaze.			o		10–15 min	
Rutter et al. (1978) Study 1	Quantitative	90% on all measures.	English university	o			9 min	
Rutter et al. (1978) Study 2	Quantitative	90% on all measures.	English university		o		9 min	
Beattie (1979)	Quantitative	91% on gaze.			o			
Goodwin (1980)	Qualitative		English transcripts	o			50 h (total)	
Lamb (1981)	Quantitative	90% on all measures.		o	o			
Harrigan and Steffen (1983)	Quantitative	93% on gaze. 85% on speech.		o			250 turns	
Harrigan (1985)	Quantitative	93% on gaze. 85% on speech.		o			14 min	
Kalma (1992)	Quantitative	83% on all measures.			o		8 min	
Egbert (1996)	Qualitative		German	o			26 h (total)	
Novick et al. (1996)	Quantitative	Transcripts checked, discrepancies discussed and corrected.	English			o	20 min (total)	
Bavelas et al. (2002)	Quantitative	90% on listener responses.			o		2 min	
Lerner (2003)	Qualitative		English transcripts	o				
Jokinen et al. (2009)	Quantitative	0.46 on all measures.	Japanese	o			4–6 min	o
Rossano et al. (2009)	Mixed	0.88 on 40% inter-rated Tzeltal data	Italian Yeli Dnye Tzeltal	o			300 sequences	
Eberhard and Nicholson (2010)	Quantitative	Gaze and speech coded by two people. Any disagreements were corrected with a third person's opinion.	American English		o		~10 min	o
Cummins (2012)	Quantitative	Overall adequate agreement	Dutch	o			15 min	
Jokinen et al. (2013)	Quantitative	0.48 on gaze0.66 on speech	Japanese	o			5 min	o
Streeck (2014)	Qualitative		American English Lebanese Arabic	o			30 conversations	
Ho et al. (2015)	Quantitative		Canadian university			o	7 min	o
Holler and Kendrick (2015)	Quantitative	0.72 on speech Gaze—transcripts checked, discrepancies discussed and corrected.	English				20 min	o
Park (2015)	Qualitative		University in U.S		o		15–30 min	
Brône et al. (2017)	Mixed	Transcripts coded by 3 people, checked, discrepancies discussed and corrected.	Dutch	o	o		15–30 min	o
Kendrick and Holler (2017)	Mixed		English transcripts	o			20 min	o
Auer (2018)	Qualitative		German	o			60 min	o
Blythe et al. (2018)	Qualitative		4 × Australian Aboriginal	o			6 h 32 min (total)	
Ijuin et al. (2018)	Quantitative		Japanese	o	o		6 min	o
Weiss (2018)	Qualitative		German	o			45–60 min	o
Zima et al. (2019)	Mixed		German and Dutch	o			302 min (total)	o

*The symbol “o” represents the feature was present*.

Only 18 of the 29 studies reported the language in which the conversations took place: two of the studies observed conversations in Dutch, three in Japanese, three in German, six in English, one in both Dutch and German, one in English and Lebanese Arabic, one in Italian, Yeli Dnye (Papua New Guinea region) and Tzeltal (Mexico region), and one study in four Australian Aboriginal languages. Of the eleven studies that that did not specify the language of the conversation, two were conducted in universities in England, one in Canada and one in US. The remaining seven studies did not specify language or location of the study (Table 4).

The studies varied in conversation activity: in 20 studies the participants were instructed to converse freely, in ten they were asked to discuss a specific topic, in two the participants completed tasks: a memory recall task (Novick et al., 1996) and a game (Ho et al., 2015) and one study (Holler and Kendrick, 2015) did not specify the instructions provided to participants (Table 4). The length of coded conversations ranged from 2 min to an hour.

All 29 studies used video recording to capture eye gaze during conversation, however nine did not specify how many cameras were used (Beattie, 1978, 1979; Rutter et al., 1978; Goodwin, 1980; Harrigan, 1985; Egbert, 1996; Lerner, 2003; Park, 2015; Blythe et al., 2018). Seven studies used one camera for each participant (Lamb, 1981; Bavelas et al., 2002; Eberhard and Nicholson, 2010; Cummins, 2012; Ho et al., 2015; Holler and Kendrick, 2015; Ijuin et al., 2018), three studies used one camera for the whole group interaction (Kendon, 1967; Harrigan and Steffen, 1983; Streeck, 2014), seven studies video recorded both each participant plus the whole group interaction (Kalma, 1992; Novick et al., 1996; Brône et al., 2017; Kendrick and Holler, 2017; Auer, 2018; Weiss, 2018; Zima et al., 2019), two studies only video recorded two out of three participants and eye tracked the third participant (Jokinen et al., 2009, 2013), one study used two cameras to capture interactions in Italian language and only one camera to capture interactions in Tzeltal and Yeli Dnye languages Rossano et al. (2009). Eleven studies used camera-based eye tracking technology (Table 4), which permits investigators to measure participant's visual behavior by detecting and tracking movement of different parts of the eye (see review: Morimoto and Mimica, 2005). Of these, two studies used a single table eye tracker to track one out of three participants (Jokinen et al., 2009, 2013) and one study tracked the eyes of two out of three participants in the conversation due to technical issues (Auer, 2018).

One study (Ijuin et al., 2018) used gaze ratio to measure the role of eye gaze in conversation, with the other 28 studies using gaze direction (Table 5). Even so, the studies largely failed to define the key variable “gaze” or defined it very vaguely (Table 5). Only five studies included a time scale in defining gaze fixation, with a starting point of gaze fixation ranging between 0.12 to 1 s (Table 5). There was also large variation in the segments of conversation analyzed including long utterances that last more than 5 s, speech interruptions, question-response sequences and backchannels (Table 5). Studies used a variety of methods to transcribe their verbal and non-verbal data; including pictographic symbols, four channel push button system, ethogram method, Conversation Analysis method (CA), GAT and GAT2 (in German language Gesprächsanalytisches Transkriptionsystem) (Selting et al., 1998) method (Table 5). Eleven studies used computer software, including Anvil, ELAN, and Adobe, to annotate their verbal and non-verbal data (Table 5). These varied methodological scenarios were examined to look for patterns in eye gaze during conversation.

**Table 5:** Definitions of key variables and coding method.

	**Definition of gaze**	**Analyzed speech**	**Coding method**
Kendon (1967)	Gaze direction	Long utterance that is ≥5 s long.Short utterances (accompaniment signals, short questions, exclamatory utterances).	Verbal and non-verbal behavior transcribed using pictographic symbols method.
Beattie (1978)	“Extended gaze” longer than 1 s. “Transient gaze” shorter than 1 s “No gaze”	One person's speech until next person starts speaking (Fries, 1952). Categorized utterances as complete, incomplete or questions (Duncan, 1972).	Speech transcribed and gaze noted at the end of utterance.
Rutter et al. (1978) Study 1	Looking behavior that causes “face reaction”	Utterance that is ≥10 words, linguistically complete, ended with turn change, and does not contain overlapping speech.	Transcribed using four-channel push-button system linked to a polygraph to visualize looking and speech.
Rutter et al. (1978) Study 2	Looking behavior that causes “face reaction”	Utterance that is ≥10 words, linguistically complete, ended with turn change and does not contain overlapping speech.	Transcribed using four-channel push-button system linked to a polygraph to visualize looking and speech.
Beattie (1979)	Gaze direction	Utterance that is ≥30 s long, linguistically complete, does not contain overlapping speech, and contain a turn yielding cue proposed by Duncan (1972). Hesitant and fluent speech classified (Beattie, 1978).	Speech transcribed and gaze noted at the end of utterance.
Goodwin (1980)	Gaze direction	Fragmented sequences consisting of restarts and pauses.	Speech transcribed in Conversation Analysis (CA) method (Sacks et al., 1974). Gaze toward the speaker indicated by a solid line.
Lamb (1981)	Gaze maintenance and gaze aversion	Speaking order.	Verbal and non-verbal behavior analyzed.
Harrigan and Steffen (1983)	Gaze measured with a reference to head direction on horizontal and vertical axes	TRP, interruptions, overlapped speech, feedback (backchannels) responses (Sacks et al., 1974).	Not specified
Harrigan (1985)	Gaze measured with a reference to head direction on horizontal and vertical axes	TRP, interruptions, overlapped speech, feedback (backchannels) response (Sacks et al., 1974).	Coding of speech was based on examples of other researchers (Yngve, 1970; Jefferson, 1973; Sacks et al., 1974) and discourse analysis. Coding of non-verbal behavior was based on examples of other researchers (Goffman, 1963; Scheflen, 1964; Kendon, 1972; Birdwhistell, 1978).
Kalma (1992)	Face gaze—looking at the eyes or surrounding area.	Action sequence (Duncan, 1983). Turn—is synonymous, not interrupted and ends with a floor change (Feldstein et al., 1979).	Verbal and non-verbal behavior transcribed using ethogram method.
Egbert (1996)	Gaze direction	Repair initiating sequence (Schegloff et al., 1977).	Speech transcribed in CA method (Sacks et al., 1974). Gaze coded using arrows in the diagrams (based on Charles Goodwin research).
Novick et al. (1996)	Gaze direction	Utterances linguistically simple, mainly containing only a name of the letter—letter sequence conversation. Turn—a period of speech without interruption.	Non-verbal behavior transcribed in detail. Speech transcribed in a narrative style (Cook, 1990).
Bavelas et al. (2002)	Measured period of mutual gaze. Gaze a fixations of 1–7 s, visual scanning between 0.25 −0.35 s (Argyle, 1967).	Listener's feedback responses.	Speaker's speech transcribed in boldface. Listener's feedback responses transcribed in italics below the speaker's speech. Mutual gaze indicated with asterisks above the speaker's speech.
Lerner (2003)	Gaze direction	Sequence initiating actions (e.g., Schegloff and Sacks, 1973)	Speech transcribed in CA method (Sacks et al., 1974). Gaze transcribed using dashed line and name initials below the speech.
Jokinen et al. (2009)	Gaze direction and length of fixations	Turn give Turn take Turn hold Turn none	Speech transcribed based on AMI corpus guidelines (www.amiproject.org; (Carletta, 2006). Non-verbal behavior transcribed based on modified MUMIN method (Allwood et al., 2007). Annotations done using Anvil program (Kipp, 2001).
Rossano et al. (2009)	Thirteen different possible gaze configurations, based on where and when the gaze was present or absent.	Question-response sequences (including: polar questions, wh- questions, alternative questions, request for new information, request for repair and request for confirmation)	Transcribed in their own developed CA method
Eberhard and Nicholson (2010)	Gaze on and off the conversation partner's face. Gaze on the face measured by two or more consecutive frames (33 ms per frame) anywhere on the face. Gaze off the face measured by one or more consecutive frames off the face (e.g., neck, torso, or wall).	The onset and offset of listener's feedback responses: Acknowledgments (e.g., mhm, hmm, oh, uh huh) Exemplifications (e.g., wow, crazy, that's weird) Request for clarifications	Speech was orthographically transcribed using Praat computer software (Boersma and Weenink, 2010).
Cummins (2012)	Looking at partner's head	Turn exchanges, Backchannels.	Speech transcribed using Praat computer software (Boersma and Weenink, 2010). Gaze transcribed based on binary distiction. Annotations done using ELAN program (Wittenburg et al., 2006).
Jokinen et al. (2013)	Gaze event—focus of visual attention on a partner or other object. Mutual gaze—focus of visual attention on each other by two conversation partners.	Turn give Turn hold Turn—a period of speech confounded by prosodic tones and pauses.	Speech transcribed based on AMI corpus guidelines (www.amiproject.org; (Carletta, 2006). Non-verbal behavior transcribed based on modified MUMIN method (Allwood et al., 2007). Annotations done using Anvil program (Kipp, 2001).
Streeck (2014)	Shifts in gaze direction. Periods of mutual gaze.	Question-response sequence Request-compliance sequence	Transcribed in their own developed CA method
Ho et al. (2015)	Beginning and end of the gaze cursor being on the conversation partner's face region.	Question-response sequence. Turn measured from start to finish of speech.	Manually coded. Annotated in Adobe Premiere Pro CS program ().
Holler and Kendrick (2015)	Gaze fixations (cursor) on conversation partners, self, surroundings, or not identified.	Question-response sequence (Stivers and Enfield, 2010). End of the turn. The first point of possible completion.	Speech segmented in Praat computer software (Boersma and Weenink, 2010). Speech transcribed based on a method by Stivers and Enfield (2010). Gaze transcribed manually on a frame-by-frame basis. Annotations done using ELAN program (Wittenburg et al., 2006).
Park (2015)	Gaze shifts	Self-repair initiating sequence.	Speech transcribed in CA method (e.g., (Sacks et al., 1974). Gaze transcribed by writing description in the brackets below the text).
Brône et al. (2017)	Brief moments of aversion and gaze fixations longer than 120 ms.	Turn take, turn hold, turn yield, turn elicit, turn complete (MUMIN; (Allwood et al., 2007). Speech segmented based on intonation units (Chafe, 1994).	Speech transcribed in GAT method (Selting, 2000) Gaze transcribed based on gaze fixations. Annotations done using ELAN program (Wittenburg et al., 2006).
Kendrick and Holler (2017)	Gaze maintenance and aversion by the respondent.	Question-response sequences (Stivers and Enfield, 2010). TCU (Sacks et al., 1974). Polar questions (Heritage, 2010).	Speech segmented in Praat computer software (Boersma and Weenink, 2010), Speech transcribed in CA method (Stivers and Enfield, 2010). Gaze transcribed manually on a frame-by-frame basis. Annotations done using ELAN program (Wittenburg et al., 2006).
Auer (2018)	Gaze direction	Sequences	Speech transcribed in GAT2 method (Selting et al., 2009) Gaze transcribed using arrows and curly brackets (Rossano, 2013).
			Annotations done using ELAN program (Wittenburg et al., 2006).
Blythe et al. (2018)	Gaze direction	Interrogatively cued question sequences, straightforward turn sequences, troubled turn sequences.	Speech transcribed using CA method (e.g., Sidnell, 2009). Gaze behavior described in a commentary text.
Ijuin et al. (2018)	Measured gaze ratio. Gaze events defined as visual attention on particular object for longer than 200 msec.	Utterances defined as segments of speech separated by pauses of more than 500 ms.	Speech transcribed manually based on start and end times of utterance. Gaze transcribed manually based on start and end times. Annotations done using ELAN program (Wittenburg et al., 2006).
Weiss (2018)	Gaze fixation and movement	Question-answer sequence Stivers and Rossano (2010).	Speech transcribed in GAT2 method (Selting et al., 2009). Gaze transcribed using circles, arrows and curly brackets (Rossano, 2012; Auer, 2018). Annotations done using ELAN program (Wittenburg et al., 2006).
Zima et al. (2019)	Gaze fixation on face region for at least 120 ms.	Overlapping speech (Fry, 1975; Walker, 2016) TCU (Sacks et al., 1974)	Speech transcribed in GAT2 method (Selting et al., 2009). Gaze transcribed manually on a frame-by-frame basis. Annotations done using ELAN program (Wittenburg et al., 2006).

### Eye Gaze

Based on the detailed examination of the articles, eye gaze patterns in conversations were grouped into six themes: (a) starting a turn, (b) eye gaze behavior during speech, (c) simultaneous speech, (d) turn yielding, (e) unaddressed participant's view, (f) unwillingness to take a turn (Table 6). Each of these is described below.

**Table 6:** A list of study outcomes.

	**Starting a turn**		**During speech**					**Simultaneous speech**	**Turn yielding**		**Sequences and courses of action**	**Unaddressed participants view**	**Unwillingness to take a turn**
	**Averted gaze**	**Direct gaze**	**Monitoring**	**Turn hold**	**Hesitant speech**	**Backchannels**	**Breakdowns and repairs**		**Averted gaze**	**Direct gaze**			
Kendon (1967)	o		o	o	o	o		o		o			
Beattie (1978)	×	×							o	×			
Rutter et al. (1978) Study 1	×	×							×	×			
Rutter et al. (1978) Study 2										o			
Beattie (1979)	×	×			o				o	×			
Goodwin (1980)			o				o						
Lamb (1981)								o					
Harrigan and Steffen (1983)		o						o		o			
Harrigan (1985)								o					
Kalma (1992)										o		o	
Egbert (1996)							o						
Novick et al. (1996)	o	o								o			
Bavelas et al. (2002)				o		o							
Lerner (2003)										o		o	
Jokinen et al. (2009)	o		o							o			o
Rossano et al. (2009)	×	×					o		×	×	o		
Eberhard and Nicholson (2010)					o	o							
Cummins (2012)	o		o			o							
Jokinen et al. (2013)			o	o						o		o	
Streeck (2014)	×	×							×	×	o		
Ho et al. (2015)	o		o							o			
Holler and Kendrick (2015)												o	
Park (2015)					o		o						
Brône et al. (2017)				o	o			o		o			
Kendrick and Holler (2017)	o	o											
Auer (2018)										o			o
Blythe et al. (2018)							o		o	o			
Ijuin et al. (2018)			o										
Weiss (2018)													o
Zima et al. (2019)								o				o	

*The symbol “o” represents the feature was present and the symbol “x” represents the feature was not present*.

#### Eye Gaze Starting a Turn

The proposal by Kendon (1967), that speakers tend to avert their gaze at the start of the turn, was confirmed in both dyadic (Cummins, 2012; Ho et al., 2015) and triadic (Jokinen et al., 2009) conversations and with acquainted (Jokinen et al., 2009; Cummins, 2012) and unacquainted (Ho et al., 2015) participants. For example, Jokinen et al. (2009) found that in 69% of cases, participants started their turn with their gaze averted from the conversation partners. Ho et al. (2015) compared eye gaze behavior relating to turn taking, during two games consisting of different rules and playing styles. The researchers found that averted eye gaze pattern at the start of the turn remained relatively consistent across both games.

When examining eye gaze behavior in relation to turn taking, Novick et al. (1996) identified two patterns of gaze behavior as two conversation partners recalled a string of letters. In this study, each group of participants were given the task of memorizing and reconstructing 17 letter sequences by taking turns in conversation. Both of the participants were given a sequence of letters. However, the sequences contained some blank spaces and only their conversation partners were able to fill those in by recalling the memorized letters. The study reported that 42% of turns had a *mutual break* pattern where speakers ended an utterance with a gaze toward the next speaker, followed by a brief mutual gaze, and then the next speaker started with an averted gaze. *Mutual hold*, which occurred in 29% of turns, differed by the next speaker holding their partner's gaze when starting their turn. The *mutual break* pattern was used more often in conversations that required fewer turn-taking attempts in order to complete the task, which associated with more successful memory recalls. The *mutual hold* was used most often in conversations requiring more attempts at turn taking, which associated with participant uncertainty about their recall and with more self or partner corrections (Novick et al., 1996). Consequently, the finding that participants were more likely to avert their gaze (*mutual break*) at the start of successful recall which imposed higher cognitive demand, suggests that eye gaze may be influenced by cognitive processing demands.

Kendrick and Holler (2017) examined eye gaze direction in relation to so-called polar questions in which you expect an affirmative “yes” or negative “no” answer and based on grammatical format of the question, the response is either preferred or dispreferred (e.g., “Can you see?”—“Yes, I can” or “Can't you see?”—“No, I can't”—*preferred* answer as positively formulated question receives positively formulated answer and vice versa for negative question. “Can you see?”—“No, I can't” or “Can't you see?”—“Yes, I can”—*dispreferred* answer because positively formulated questions receive negatively formulated answer and vice versa for negatively formulated question; see: Bolden, 2016; Kendrick and Holler, 2017). Kendrick and Holler (2017) found that 53.8% of responses to polar questions started with a speaker gazing away. However, speakers responded to the majority of preferred responses with a gaze directed at the questioner, except when giving complex responses or when taking time to think about their response, when they kept their gaze averted. Furthermore, the majority of dis-preferred listener responses were produced with gaze averted.

The latter two studies do not provide a strong case for Kendon's (1967) claim that speakers tend to gaze away at the start of the turn, with only 42% (Novick et al., 1996) and 53.8% (Kendrick and Holler, 2017) of turns starting with averted speaker's gaze. However, both studies suggest that in the cases when speakers avert their gaze at the start of a turn, this gaze pattern may be related to the level of cognitive processing with more complex responses requiring more planning and concentration, during which speakers avert their gaze. Additionally, Harrigan and Steffen (1983) found no supporting evidence that speakers avert their gaze at the start of the turn. The researchers analyzed eye gaze patterns of five people in a group conversation and found that 79% of the time, the speakers tended to gaze toward a listener at the start of the utterance (Harrigan and Steffen, 1983).

Finally, four other studies (Beattie, 1978; Rutter et al., 1978; Rossano et al., 2009; Streeck, 2014) concluded that their evidence did not support the suggestion that eye gaze facilitates turn taking. Instead, two of these studies Rossano et al. (2009), Streeck (2014) argue that eye gaze is used to coordinate an initiation, formation and closure of action sequences (e.g., question-answer, request-compliance, telling-appreciation) that may take multiple turns to complete (Rossano et al., 2009). More specifically, Rossano et al. (2009) investigated question-answer sequences extracted from three different cultures speaking different languages and found that only a small proportion (Tzeltal−10.7%, Yeli Dnye−12.3%, Italian−16.4%) of questions were asked by the speaker with averted gaze. In contrast, the speakers were more likely to initiate the sequence by gazing toward the recipient (Rossano et al., 2009; Streeck, 2014). Streeck (2014) argued that speaker's gaze on the listener, whether they maintain the gaze during the question initiation or bring the gaze back from another preoccupying task (e.g., eating) during the sequence, is to indicate the salience of what is being said. Furthermore, gazing at the listener during question initiation, allows the speaker to check that the listener understands, believes and/or agrees with the intentions of the act rather than merely checking if the listener is paying attention (Streeck, 2014).

#### Eye Gaze During Speech

In line with Kendon (1967), several studies confirmed that eye gaze has a monitoring role during conversations (Eberhard and Nicholson, 2010; Cummins, 2012; Jokinen et al., 2013; Ho et al., 2015). In general, listeners tend to gaze at the speaker for long periods of time (Kendon, 1967; Cummins, 2012; Jokinen et al., 2013; Ho et al., 2015), whereas speakers gaze less often and give regular, short glances toward the listener (Kendon, 1967; Eberhard and Nicholson, 2010; Jokinen et al., 2013; Ho et al., 2015). This phenomenon has been observed in dyadic (Kendon, 1967; Eberhard and Nicholson, 2010; Cummins, 2012; Ho et al., 2015), triadic (Jokinen et al., 2013) free flowing (Kendon, 1967; Cummins, 2012; Jokinen et al., 2013), storytelling (Eberhard and Nicholson, 2010) and game context (Ho et al., 2015) conversations. However, an opposite effect has been observed during question-answer sequences (Rossano et al., 2009; Streeck, 2014). For example, Rossano et al. (2009) explored gaze behavior in three different cultures and found that speakers tend to gaze toward the listener more often (Tzeltal−65.7%, Italian−79.9%, Yeli Dnye−79.9%) than listeners toward the speaker (Tzeltal−42.3%, Italian−63.3%, Yeli Dnye−67.3%).

Ijuin et al. (2018), examined the role of eye gaze in relation to turn taking within groups of three native or non-native language speakers. This is the only study that looked at the amount of time spent gazing, rather than gaze shift patterns, to predict turn taking. They found that speakers in both native and non-native speaking groups tend to look more at the person who is likely to be the next speaker than at the observing listener, in both *floor- switching* and *floor-holding* conditions. Furthermore, speakers in both language groups looked at the next speaker more in *floor-switching* than *floor-holding* conditions (Ijuin et al., 2018), suggesting that gazing ratio between three conversation partners can be used to predict the next speaker.

“Backchannels”—the short verbal and non-verbal signals used by listeners to acknowledge the speaker and to convey their understanding—were found to be elicited during mutual gaze between a listener and a speaker (Kendon, 1967; Bavelas et al., 2002; Eberhard and Nicholson, 2010; Cummins, 2012). However, Eberhard and Nicholson (2010) found a slight difference between verbal and non-verbal backchannels and the occurrence of mutual gaze. The findings suggest that overlap between mutual gaze and backchanels were more likely to happen during listeners' non-verbal signals, (e.g., acknowledgments—head nods; and exemplifications—facial expressions; 80 and 93% respectively), than verbal signals (i.e., acknowledgments—“ok,” “mhm,” “uh huh”; exemplifications—“wow, that's crazy”; 60 and 69%, respectively). The findings suggest that verbal signals alone help to convey listener's engagement and understanding, therefore speakers are less inclined to visually check upon the listeners.

In their studies, Jokinen et al. (2009, 2013) found that seating positions have a significant effect on how participants in triadic conversations divide their visual attention between conversation partners. They found that a participant sitting in front of two partners, divided their attention between them equally. However participants with one partner in front of them and another to the side, spent about 45% of the time gazing in the distance, 40% of the time at the partner in front of them, and only 15% of the time to the partner sitting next to them (Jokinen et al., 2009). These findings suggest that seating position may mediate the effectiveness of eye gaze ratio in predicting the next speaker, where the seating arrangements are not equally distributed.

In relation to breakdowns in conversations, Goodwin (1980) found that in order to produce a coherent sentence, speakers preferred to have recipient's gaze secured. They found that during conversation breakdown, speakers restarted their sentence as a technique to request the listener for their gaze. Goodwin (1980), suggested that in order to avoid restarts, it is preferred that the listener is gazing at the speaker when the speaker looks at the listener and not the other way around. Furthermore, the speakers also used pauses near the beginning of the sentence to delay speech until the listener's gaze was obtained (Goodwin, 1980; Streeck, 2014). Egbert (1996) found that in all of the segments containing the repair-initiator “pardon?” (“bitte?” in German), the speaker did not share mutual gaze prior to initiation of repair. Whilst, Rossano et al. (2009) found that repair-initiating questions were often initiated with a mutual gaze between speaker and the listener. However, Goodwin's (1980) suggestion that speakers preferred to have recipient's gaze secured to prevent conversation breakdown, were not supported by Rossano et al. (2009), who found that during 20% to 30% of questions the listener's gaze was not present and repairs were not initiated as a result. In contrast, Streeck (2014) found evidence that when the speaker's gaze was not present during the question, it led to the recipient of the question failing to respond. Blythe et al. (2018) found that during problematic next speaker selection, when the intended addressee in multiparty conversation fails to respond, problems often arose due to seating arrangements and lack of mutual gaze. Blythe et al. (2018) noted that when the addressee fails to respond, the current speaker tends to use more engagement tools than before, such as turning their head to gaze toward the addressee or make vocative reference such as calling the person's name.

A consistent pattern of averted eye gaze during hesitant speech has been found. Kendon (1967), reported that speakers looked at the listeners around 50% of the time during fluent speech, but only 20.3% of the time during hesitant speech. Beattie (1979) found that hesitant speech which requires more planning (i.e., cognitively challenging) was associated with averted gaze. Park (2015) found that in interactions with teachers, students were more likely to use an “or-prefaced” self-repair sequence (i.e., immediately starts another turn with an “or” to give an alternative example), when teachers used dispreference signals, such as hesitation and pauses, which was often accompanied by a teacher's eye gaze shift from a mutual eye gaze. Two studies (Eberhard and Nicholson, 2010; Brône et al., 2017) found that speakers avert their gaze away from the listener during verbal pause fillers (e.g., “uhm”), a behavior associated with speech planning Eberhard and Nicholson (2010). More specifically, Brône et al. (2017) found this gaze pattern in 76% of cases and Eberhard and Nicholson (2010) reported this pattern in six out of seven speakers. Similarly, four other studies found that speakers do tend to terminate their gaze to indicate the turn hold (Kendon, 1967; Bavelas et al., 2002; Jokinen et al., 2013; Zima et al., 2019), which happens at Transition Relevance Place (TRP: Table 2), during switching pauses and hesitant markers (Jokinen et al., 2013; Brône et al., 2017). Jokinen et al. (2013), concluded that eye gaze direction was a better predictor of turn hold than speech.

Finally, evidence suggests that eye gaze behavior tends to vary between and within conversations. For example, Cummins (2012) examined individuals' behavior in multiple dyadic conversations with different partners, and found that gaze varied between conversations, suggesting (i) eye gaze behavior is adaptive and (ii) likely to be influenced by the behavior of the conversation partner. Streeck (2014) also found that speaker's gaze toward the listener varied in frequency and duration, however unlike Cummins' study, variation was higher within rather than between conversation. It is important to note that Streeck's (2014) findings were based on one speaker's gaze rather than multiple speakers as in Cummins' (2012) study, as such, this may help to explain the difference between these studies. Rossano et al. (2009) found that amount of gaze varied based on the type of question sequence and its position in the sequence. For example, speakers tend to gaze less during request for information that mostly occur at the start of the sequence, than during request for repair and confirmation sequences that mostly occur within an initiated sequence (Rossano et al., 2009). There is also evidence to suggest that amount of gaze tend to vary between cultures, with some cultures (i.e., Italian and Yeli Dnye) gazing toward the conversation partner more than in others (i.e., Tzeltal; Rossano et al., 2009).

#### Eye Gaze During Simultaneous Speech

When it comes to simultaneous speech, Schegloff (2001) noted that interruptions can be classed as problematic and unproblematic. Schegloff (2001) defined, *problematic interruption* is when the listener disrupts the speaker's speech with the aim of taking the floor, which prevents the other person finishing their turn. In contrast, *unproblematic overlap* is a short period of simultaneous speech where one speaker is finishing their turn and another is starting their turn prematurely (Schegloff). In this review, six studies (Kendon, 1967; Lamb, 1981; Harrigan and Steffen, 1983; Harrigan, 1985; Brône et al., 2017; Zima et al., 2019) examined eye gaze behavior during problematic and unproblematic speech interruptions and identified two different situations: *starting an initial interruption* and *prevailing the interruption* once the interruption has started.

Three studies (Kendon, 1967; Harrigan and Steffen, 1983; Brône et al., 2017) reported a similar gazing pattern at the start of initial interruption. Despite a small number of interruption occurrences, Kendon (1967) observed that during problematic interruption, speakers tend to stare at each other, until one prevails. Harrigan and Steffen (1983) found that interrupting speakers gazed at the listeners at the start of 90% of successful and 83% of unsuccessful problematic interruptions, and 63% of the time at the start of unproblematic overlapped speech. Brône et al. (2017) investigated dyadic and triadic conversations, and found that individuals wishing to interrupt the speaker, often averted their gaze prior to problematic interruption and then mostly started the interruption with a direct gaze at the interrupted speaker.

Two studies (Harrigan, 1985; Zima et al., 2019) also found a similar eye gaze pattern that influenced one of the speakers to prevail at the interruption once the interruption had started. Harrigan (1985) examined verbal and non-verbal behavior in relation to turn-taking and found that gazing away was a strategy in prevailing at problematic interruption. Zima et al. (2019) found that during simultaneous speech, in 54.7% of cases with a mutual gaze, speakers who averted their gaze first, won the competition for a turn take, and 80.5% of these speakers completed their turn successfully, whether that was a turn-holding (problematic interruption) or turn-yielding (unproblematic overlap) scenario. Furthermore, in 62.1% of interruption cases without mutual gaze, the speaker who gazed at the other speaker, lost the competition for the turn, whereas in 75.8% of cases where the speaker avoided another speaker's gaze, won the competition.

Finally, Lamb (1981) examined gender difference regarding dominance and speaking order in same sex triads and found that females who simultaneously spoke first were more likely to avert their gaze, whereas males in the same situation tended to maintain their gaze.

#### Eye Gaze During Turn Yielding

In line with Kendon's (1967) study, 11 studies confirmed that in general people tend to end their turn with eye gaze directed at the next speaker (Rutter et al., 1978; Harrigan and Steffen, 1983; Kalma, 1992; Novick et al., 1996; Lerner, 2003; Jokinen et al., 2009, 2013; Ho et al., 2015; Brône et al., 2017; Auer, 2018; Blythe et al., 2018). Kendon (1967) reported that around 71% of speaker turns ended with a gaze toward the listener and 69% did so in Harrigan and Steffen's (1983) sample. As mentioned above, Novick et al. (1996) identified “mutual break” and “mutual hold” patterns, which between them a total of 71% of turns ended with a gaze toward next speaker. Auer's (2018), study confirmed that people end their turn with a directed eye gaze, but highlighted that gaze is not always the dominant factor in selecting the next speaker. For example, where a speaker addresses a generic question (e.g., to identify a location) to two listeners, but only addresses one of the listeners by gaze. However, if a gaze-addressed individual is taking their time to answer, for example when they are not sure of the answer, the second, gaze-unaddressed participant, who knows the answer or gathers their thoughts faster, is likely to take the turn.

Kalma (1992) investigated the role of prolonged gaze, which was linked to participants being more dominant in triadic conversations and found that listeners who received a prolonged gaze from the speaker at the end of the utterance, were most likely to be the next speaker. Blythe et al.'s (2018) study highlighted the importance of using direct eye gaze and other engagement tools, such as head turns or vocative references in order to achieve unproblematic next speaker selection, in which the speaker selected next by the current speaker takes a turn. However, Blythe et al. (2018) also found that during non-selecting interrogative questions in multiparty conversations, in which no one is being addressed, the current speaker gazed away from all the listeners to avoid selecting the next speaker, suggesting the direction of eye gaze in turn taking can be context specific. Rutter et al.'s (1978) second study found that speakers tended to gaze at the end of the utterance more for strangers than friends. Similarly, speakers at the end of the utterance were less likely to gaze during a cooperative topic about socio-politics (i.e., held the same point of view), than a competitive topic (i.e., held opposite points of view) (Rutter et al., 1978).

As mentioned before, four studies (Beattie, 1978; Rutter et al., 1978; Rossano et al., 2009; Streeck, 2014) concluded that their evidence did not support the suggestion that eye gaze facilitates turn taking, with two studies Rossano et al., 2009; Streeck, 2014 claiming that eye gaze instead was used in relation to organization of action sequences that may take multiple turn to complete. Nevertheless, Streeck (2014) did find that the recipient of the question, is more likely to respond if the speaker is gazing at the recipient at the end of the question. In response to Kendon's (1967) finding that speakers tend to look at the listener at the end of the turn, Rossano et al. (2009) analyzed question-answer sequences from three different language samples and found that speakers at the end of the question very rarely broke and shifted their gaze back to the listener at the end of the turn (Tzeltal−7.7%, Yeli Dnye−5%, Italian−5% of cases). Rossano et al. (2009) argued that due to the fact that speakers tend to look at the listener throughout the question without shifting their gaze, it cannot be used as a cue for switching speaker roles. However, when it comes to the end of question-answer sequence, both the listener and the speaker indicate closure by gazing away from one another (Rossano et al., 2009; Streeck, 2014). Similarly, Weiss (2018) who investigated eye gaze behavior in relation to turn taking in triadic conversations, reported that in the instances when no one had anything to say, the topic was closed by all three conversation partners gazing away from each other.

#### Eye Gaze and Unaddressed Participant

The role of unaddressed participant's eye gaze within conversation has been noted and discussed in several studies (Kalma, 1992; Lerner, 2003; Jokinen et al., 2013; Holler and Kendrick, 2015; Zima et al., 2019). Kalma (1992) examined the function of “prolonged” eye gaze in relation to turn taking and found that unaddressed participants were less likely to interrupt speech, if the next speaker was selected with a prolonged gaze. Lerner (2003) investigated how speakers select the next speaker in multi-party conversations and found that problems such as speech interruption occur when an unaddressed participant does not see the speaker's intentions and takes the turn instead. Jokinen et al. (2013) noted that in triadic conversations, the observing recipient gazed at the current speaker less than the primary addressee who is being addressed by the speaker, which increased the likelihood that the primary addressee would be the next speaker. Holler and Kendrick (2015), explored the timing in relation to turn taking from the unaddressed person's perspective in triadic conversation. They found that during question-answer sequences, the unaddressed participant most often shifted their gaze to the next speaker 50 ms prior to the anticipated end of their turn, and 40 ms prior to the first point of passible completion of question turns. Holler and Kendrick (2015) concluded that the gaze of the unaddressed participant is mostly anticipatory, but they are also sensitive to TRP cues (Sacks et al., 1974). Zima et al. (2019) found that during speech interruption, in 60.2% of the cases, the unaddressed participant helped to appoint the next speaker by gazing either at the original speaker or the speaker who interrupted the speech.

#### Eye Gaze With Partner Unwilling to Take a Turn

Three studies (Jokinen et al., 2009; Auer, 2018; Weiss, 2018) found that the gaze-selected next speaker (i.e., the current speaker is gazing at the person they expect to speak next) who either does not know how to respond or is just unwilling to take a turn can decline the offer by averting their eye gaze from the current speaker. However, this phenomenon has only been tested in triadic studies (Jokinen et al., 2009; Auer, 2018; Weiss, 2018), and observed during question-answer sequences (Auer, 2018; Weiss, 2018), so it is unclear if the same would apply to dyadic conversations and during different type of turn sequences. Weiss (2018) also found that in 56% of cases, a gaze-selected next speaker was able to pass on the turn intended for them, by redirecting their gaze to the unaddressed participant in a triad. In the instances when no one had anything to say, the topic was closed by all three conversation partners gazing away from each other (Weiss, 2018).

## Discussion

The aim of this review was to investigate the literature on the role of eye gaze in relation to turn taking and how this had been studied over the last 50 years. Six themes describing the role of eye gaze in conversation were identified based on 26 studies carried out between 1967 and 2019. Specifically, these themes related to the function of eye gaze at the start of a turn, during conversation, during speech interruption and overlap, at the end of the turn, eye gaze from the view of an unaddressed participant, and finally the role of eye gaze when a participant is unwilling to take a turn.

### Eye Gaze

During conversation, people use eye gaze to monitor each other's availability, reactions and emotions (Kendon, 1967; Eberhard and Nicholson, 2010; Cummins, 2012; Jokinen et al., 2013; Ho et al., 2015). Listeners tend to gaze at the speaker more and for longer periods to show their interest, whereas speakers tend to gaze at the listeners more frequently, but for a shorter period of time to monitor listener's focus of attention. In support, Argyle and Dean (1965), Argyle et al. (1973) argued that people who were able to see their conversation partners spent more time looking at them to seek additional information, than the participants who were unable to see their conversation partners but knew the location they were seated. In other words, given the opportunity, people prefer to observe their interaction partner. However, the fact that speakers spend less time gazing suggests that direct eye gaze may be distracting for the speaker and averted eye gaze may be needed for continuous speech planning or perhaps to avoid being interrupted by the listener by showing their unavailability. Another two studies (Rossano et al., 2009; Streeck, 2014) found an opposite effect that during question-answer sequences speakers tend to gaze more toward the listener than other way around. One explanation for this may be to do with the fact that studies differed in types of conversation they analyzed. As reported in Rossano (2012) thesis, gaze behavior of the listener tends to differ when listening to stories vs. simple questions. However, Ho et al. (2015) also analyzed question-answer sequences, but the results supported Kendon's claim. Ho et al. (2015) study was conducted in a game context rather than free flowing conversation, carried out in a laboratory setting rather than in a natural environment and analyzed using statistical method rather than CA. This highlights that gaze behavior is not straightforward and is influenced by a combination of factors. Furthermore, monitoring each other during conversation is important for coherent conversation, as the presence of mutual gaze helps to avoid conversation breakdowns (Goodwin, 1980; Egbert, 1996; Blythe et al., 2018) and is often used to restore the breakdowns (Rossano et al., 2009; Streeck, 2014). In addition, monitoring each other during conversation helps to prompt backchannels (Kendon, 1967; Bavelas et al., 2002; Eberhard and Nicholson, 2010; Cummins, 2012), which are used to show listener's understanding and focus of attention, and also help the speaker to tell a story with more enthusiasm, dramatic endings and without repetition (Bavelas et al., 2000, 2002; Bertrand et al., 2007).

A specific eye gaze pattern has been observed during speech interruptions when the listener disrupts the speaker's speech with the aim to take the floor (Kendon, 1967; Harrigan and Steffen, 1983; Brône et al., 2017). The mutual eye contact (Kendon, 1967) or gazing at the interrupted speaker at the initial start of the simultaneous speech (Kendon, 1967; Harrigan and Steffen, 1983; Brône et al., 2017) may function as a way to check the conversation partner's reaction. Whereas looking away to break the mutual gaze once the interruption has started, to win over the turn, may signal that person's unavailability to accept further information from the other speaker (Harrigan, 1985; Zima et al., 2019) and signal commitment to speech planning (Glenberg et al., 1998). In fact, shifting one's eye gaze at TRP from the conversation partner has also been linked to a floor-holding strategy when people need time to gather their thoughts of what they going to say next (Kendon, 1967; Bavelas et al., 2002; Jokinen et al., 2013; Zima et al., 2019). Overall, there seems to be similar eye gaze behavior of looking away, whether that is when aiming to hold the floor to continue talking or interrupting speech to start an abrupt turn, all of which are likely linked to the cognitive processes involved in speech planning or to indicate speaker's unavailability to receive a response from a listener.

Furthermore, eye gaze behavior of the unaddressed participants in multiparty conversations appears to play a large role in monitoring and managing conversations (Jokinen et al., 2009; Auer, 2018; Weiss, 2018), by contributing to a prevention of simultaneous speech or by helping to solve a dispute between two conversation partners who speak simultaneously in competing for a turn (Lerner, 2003; Zima et al., 2019). By monitoring conversations, the unaddressed participants are able to perceive each partners' intentions and help to keep the conversation going smoothly. However, the unaddressed participant who is not paying full attention to conversation partners, can equally be the ones interrupting the speech (Lerner, 2003). In addition, evidence suggest that unaddressed participants are able to anticipate the end of the turn and tend to shift their gaze to the next speaker prior to the end of the turn, or at least in question-response sequences (Holler and Kendrick, 2015). This provide evidence that unaddressed participants are in tune to listen out for TRP cues in order to ensure a smooth transition between the speakers (Holler and Kendrick, 2015).

When it comes to the end of the turns, the studies reported in this review, strongly support Kendon's (1967) findings that individuals are likely to look at their conversation partner at the end of the turn (Kendon, 1967; Rutter et al., 1978; Harrigan and Steffen, 1983; Kalma, 1992; Novick et al., 1996; Lerner, 2003; Jokinen et al., 2009, 2013; Ho et al., 2015; Brône et al., 2017; Auer, 2018; Blythe et al., 2018; Streeck, 2014) to check the next speaker's availability and to signal turn yielding. Direct eye gaze at the end of the turn is especially important in multiparty conversations, as direct eye gaze is often used to select the next speaker (Blythe et al., 2018). However, Rossano et al. (2009) argued that a claim that participants return their gaze to the listener as a way to invite them to take a turn does not apply to their findings of question-answer sequences, because the speaker tends to look at the listener throughout the question without shifting their gaze. As such eye gaze cannot be used as a cue for switching speaker roles. However, one could argue the fact that the speaker asking a question, with raised intonation at the end (Duncan, 1972), while gazing toward the recipient, is a cue for them to take the floor.

The findings relating to eye gaze behavior relating to the start of the turns are less consistent. Kendon's (1967) claim that speakers tend to avert their gaze at the start of the turn has been supported by three studies (Jokinen et al., 2009; Cummins, 2012; Ho et al., 2015) and disputed by five (Beattie, 1978; Rutter et al., 1978; Harrigan and Steffen, 1983; Rossano et al., 2009; Streeck, 2014). It is difficult to pinpoint one reason for different findings, as there are multiple methodological factors which could have influenced the results. However, one interesting point is that two studies (Rossano et al., 2009; Streeck, 2014) claimed that rather than facilitating turn taking as such, eye gaze instead plays a role in the organization of action sequences. This alternate approach to the role of eye gaze was reported more than a decade ago, but has not been studied as extensively as Kendon's original claim and there are still many questions to answer. For example: what role does eye gaze have in relation to other types of action sequences? How does it function in triadic conversation settings? what role does the unaddressed participant have? and would the findings be the same if the study was conducted in a controlled laboratory environment without interfering activities and objects? It appears that eye gaze does play a role in communication, however, as reported in this review, not all turns, nor all question-answer sequences started or ended with a predicted gaze, suggesting that other factors may also contribute. Future studies, may benefit from analyzing and reporting on those specific cases to determine what influenced speaker and listener behavior.

Harrigan and Steffen (1983) also found different results from Kendon, however they were the only researchers reported in this review attempting to study eye gaze behavior in a larger group situation. Future studies should explore eye gaze behavior in different group sizes to see how it differs with increasing number of participants. The findings of two other studies are indecisive regarding Kendon's (1967) claim, as only 42% (Novick et al., 1996), and 53.8% (Kendrick and Holler, 2017) of the time the speakers averted their gaze at the start of the turn. However, both studies (Novick et al., 1996; Kendrick and Holler, 2017) indicated that averted eye gaze at the start of the utterance is linked to cognitive processing of speech, with aversion linked to more complex cognitive demands. This interpretation is in line with Glenberg et al. (1998) findings that individuals answering difficult questions requiring more cognitive processing were more likely to avert their gaze before responding. Furthermore, individuals who were instructed to fixate their gaze during a difficult recall task, performed worse than participants who were able to avert their gaze (Morales et al., 2000). This cognitive processing idea may help to explain some predicted gaze discrepancies within the literature, as perhaps the easier questions and/or responses does not need much planning and concentration.

### Study Design

Kendon (1978) originally proposed that different findings in relation to gaze and turn taking, may be due to difference in study designs. In this current review, no two studies used the same design and many lacked the essential details needed for replication or comparison with other studies. First and most importantly, the majority of studies reviewed failed to provide their definition of “eye gaze.” The few studies that did, gave different definitions. For example, Rutter et al. (1978) defined eye gaze as “looking” behavior that caused a “face-reaction.” This definition is quite vague as it does not explain different looking behavior or what “face-reaction” means. Jokinen et al. (2013) defined “gaze event” as a focus of visual attention but failed to define what exactly “focus” means and how long it lasts. Brône et al. (2017) defined “gazed” as fixation of anything longer than 0.12 s. Ijuin et al. (2018) defined gaze as “visual attention longer than 0.2 s.” In addition, Bavelas et al. (2002) reported that visual scanning lasts around 0.025 to 0.35 s and gaze fixation is longer than 1 s. It is unclear what time restrictions other studies used to define gaze. The continued existence of such variations is somewhat surprising given that back in 1978, Beattie (1978) criticized Kendon for not defining the key variables in his 1967 study and was the first person to define gaze in relation to duration. However, despite this information being available, only a handful of studies have taken this into consideration.

A failure to clearly define eye gaze and the use of different definitions in different studies both contribute to different studies reporting different findings. These differences extend to definitions of turns and types of exchange sequences (Table 5). For example, the earlier, pre-1980 studies were more likely to use their own definitions such as utterances longer than 5 s (Kendon, 1967) or utterances longer than 10 words that ended with a floor change (Rutter et al., 1978). Some later studies adopted the better-defined approaches that used TRP and exchange sequences to define turns (e.g., Sacks et al., 1974; Stivers and Enfield, 2010; Table 5). Hence, the findings from studies that used different definitions or turn sequences, may not generalize to other types of turns. Furthermore, as mentioned above, Rossano (2012) analysis of dyadic conversations revealed that participants' eye gaze behavior was most likely to be influenced by sequential organization of the turns proposed by Schegloff and Sacks (1973), Schegloff (1972), Schegloff (1997), Sacks et al. (1974). For example, listeners' eye gaze patterns are different when they are listening to simple questions, instructions or remarks than when they are listening to extended stories. This may further explain the reason why not all turns and sequences start or end with a predicted eye gaze pattern (Rossano, 2012).

The majority of pre-2002 studies tended to use same-sex only conversation, with mixed sex conversations coming in later studies. Some studies used a selection of same sex and mixed conversations, however only two studies directly compared gender. Rutter et al. (1978) found no difference in eye gaze behavior between male only and female only conversations, whereas Lamb (1981) found that females who simultaneously spoke first were more likely to avert their gaze, while males tended to maintain their initial gaze. It is surprising that the majority of studies reviewed here ignored gender difference in eye gaze patterns when designing their studies, as these were documented in several early papers (Argyle and Dean, 1965; Myszka, 1975; Bissonnette, 1993). Myszka (1975) noted that during interviews, female interviewees maintained eye contact more than males. Furthermore, participants in same sex interviews, appeared more anxious than participants in mixed sex interviews, which resulted in low levels of eye contact. Bissonnette (1993) found that females shared more mutual gaze in the same sex dyads than males did, suggesting female preference for a higher level of intimacy.

Referring back to Beattie's (1978, 1979) studies, the analyzed dyads were male only and consisted of status-influenced conversations between students and supervisors. This closely resembles Myszka's (1975) study in which male only dyads were also influenced by status between interviewer and interviewee, reporting low levels of eye gaze. As mentioned before, the social status of one conversation partner affects eye gaze behavior of the other conversation partner, such that people with a high status tend to be observed more often and for a longer periods of time, than people with a lower status (Foulsham et al., 2010). As such, Beattie's (1978, 1979) may have been influenced by both gender and status factors that resulted in non-significant results. However, Beattie's study outcomes should not be dismissed, even if conversant's status or a combination of variables prove to change eye gaze behavior, as it still adds knowledge on how different variables within conversations change eye gaze behavior. Future research should investigate eye gaze behavior in relation to turn taking in conversations influenced by status differences between conversation partners.

Age and participants' ethnic background are two further variables that studies in general did not report. To our knowledge, there is no supporting literature that eye gaze patterns during turn taking tend to vary in the adult population based on age, but it would be a useful information to use to generalize results or to compare them. Participants' ethnic background is another important variable, as there is evidence that eye gaze patterns can differ across ethnic groups (LaFrance and Mayo, 1976) and cultures (Li, 2004; McCarthy et al., 2006; Rossano et al., 2009). LaFrance and Mayo (1976) found that black individuals spent less time looking at conversation partners during listening, but more time during speaking, and an opposite effect was found for white participants. Li (2004) found that in Canadian/Canadian dyadic conversation, participants were gazing at their partner more often and for longer periods of time, than participants in Chinese/Chinese dyadic conversation. McCarthy et al. (2006) reported that when having to think about cognitively demanding mathematical, verbal or spatial questions, Japanese participants were most likely to look down, whereas Canadians and Trinidadians were most likely to look up. However, when answering easy questions, the Japanese participants again were most likely to avert their gaze, whereas Trinidadians most often gazed directly and maintain mutual contact. The studies in this review were conducted in a variety of languages (Table 4). Whilst the results confirm similar findings across some of these languages (e.g., English, German, Dutch, Japanese, and Australian Aboriginal), Rossano et al. (2009) found some cultural gaze variation in a sample studying Italian, Tzeltal and Yeli Dnye languages. Therefore, it is not possible to confirm that gaze behavior would generalize across all languages and cultures.

Only four of the reviewed studies (Kendon, 1967; Beattie, 1979; Lamb, 1981; Novick et al., 1996) reported physical distance between participants, and these all differed, ranging from three to six feet. Other studies either did not report any information or stated that participants sat across a table, even though evidence that distance between conversation partners has an effect on eye gaze behavior was an early finding (Argyle and Dean, 1965; Argyle et al., 1973). Argyle et al. (1973) found that people sitting two feet apart felt most uncomfortable and shared the least amount of eye contact and that eye contact increased with distance. This was most prominent for the opposite sex pairs, suggesting that eye gaze is a cue for intimacy (Argyle and Dean, 1965; Argyle et al., 1973). This is a very important variable that the majority studies reviewed here did not report.

Another potentially influential factor mentioned earlier, is conversation topic. Studies in this review reported free flowing conversations, memory recall tasks and discussion on a specific given subject, all of which differed in speech complexity and required different levels of cognitive processing. Early evidence suggested that when participants are asked very personal questions about their fears and desires, they are more likely to avoid mutual gaze with an interviewer than during non-personal questions (Exline et al., 1965). Further evidence suggests that eye gaze behavior differs during cooperative and competitive conversations, with speakers at the end of the turn gazing less during a cooperative, than competitive topic (Rutter et al., 1978) or more likely to avert their gaze when answering more difficult than and easy questions (Glenberg et al., 1998). Kendon (1967) also noted that the amount of mutual gaze tends to decrease with an increase of high emotion (i.e., smiling) during conversations. These are interesting findings and future studies could benefit from reporting the general mood of participants or the tone of conversation for further analyses or comparison between studies. In the current review, the authors were unable to compare the studies based on the topics, because most studies only reported that the conversation was free flowing, or the studies that used specific topics did not specify what the tone of those conversations were. Furthermore, the decision making behind selecting the topic and type of conversation was mainly missing in the papers reviewed.

Acquaintance status (i.e., known or unknown conversants) is another design decision which was frequently not explained or fully explored. Strongman and Champness (1968) found that unacquainted participants that shared positive mutual affiliation spent significantly more time speaking with direct gaze at the partner. Rutter et al. (1978) concluded that acquainted participants gaze less at the end of utterances, whereas Bissonnette (1993) noted that friends in general gazed at each other more, that unacquainted participants. Further evidence suggests that couples who are in love, gaze at each other more (Rubin, 1970). It appears that eye gaze patterns may differ based on a level of affiliation between conversation partners (Strongman and Champness, 1968; Rubin, 1970), but other factors can also influence this.

There was also lack of consistency and reporting regarding sample sizes and effects. The quantitative studies we reviewed reported sample sizes between 5 and 69 participants and also different lengths of video segments ranging from 2 min to 1 h. However, none of these studies explained their reasons for their sample size or more importantly reported any effect size of their findings. Among qualitative studies, some did not report the number of participants or how many examples were analyzed to reach their results. The majority of the quantitative studies reported reliability scores, which mostly were highly reliable. However, none of the qualitative and only one mixed design study conducted reliability checks. Whilst qualitative studies do not use statistical methods to establish reliability, there are a variety of ways to enhance trustworthiness of study findings, such as discussing and seeking agreement with another person (Noble and Smith, 2015). Therefore, it is possible that qualitative studies reported here could be influenced by subjective bias.

The studies summarized here also differed in approaches they chose to analyze the data, with a majority of studies opting for a statistical approach (i.e., quantitative studies) where they analyzed number of gaze occurrences at the start and the end of the turns. Others opted for the CA approach (i.e., all qualitative and mixed method studies), which looks at the bigger picture of interaction, by taking human actions and social context into consideration (Goodwin and Heritage, 1990). Both approaches have their strengths and weaknesses. For example, the statistical approach allows the researchers to explore specific hypotheses and objectively analyze data using statistical methods. However, a statistical approach does not allow much exploration beyond the hypothesis (Queirós et al., 2017). In contrast, CA does not focus on specific predictions but explores the subject by taking overall context into consideration (Van Tam, 2016; Queirós et al., 2017). When it comes to studying language, context is very important to understand real meanings and intentions (Van Tam, 2016). However, the CA method is prone to subjectivity bias to researcher's point of view (Queirós et al., 2017).

Another potentially influential factor to consider is the setting of the studies. Most studies reported here (Table 3) were conducted in a controlled laboratory setting, with limited distractions. In contrast, the ecological studies (Table 3) were likely influenced by different seating arrangements, participants moving around, handling objects, or being distracted by environmental factors (e.g., Goodwin, 1980; Rossano et al., 2009; Blythe et al., 2018; Streeck, 2014). Again, both approaches have their strengths and weaknesses. The laboratory-based studies were able to control influential factors resulting in arguably more concrete results. However, these findings do not necessarily generalize directly to real life scenarios. In contrast, the ecological studies explored gaze behavior in a natural setting. However, the uncontrolled environmental variables may have affected gaze behavior, resulting in different conclusions.

In terms of coding, 16 studies reviewed here, coded their data manually, that is eye gaze direction was determined by the researchers. The remaining ten, mainly post-2003 studies, used eye tracking devices (Table 4), which is likely to be more accurate, as it is not influenced by interpretation bias. However, eye tracking studies are susceptible to data loss due to technical and calibration issues, which was observed in a few studies review here (Jokinen et al., 2009, 2013; Ho et al., 2015; Holler and Kendrick, 2015; Auer, 2018). Furthermore, wearing an eye tracking device may influence eye gaze behavior, as participants are aware that the study is looking into eye gaze behavior even if the researchers did not inform them of the true intentions. Another issue is regarding accuracy of coding mutual gaze. It is difficult to determine a precise place on the face where participants were looking (i.e., eyes, lips, forehead) when coding was done manually. However, eye tracking studies have precise information but often did not to report if mutual gaze was the only measure when participants were looking each other directly in the eye, or whether slight deviation from the eye region was also coded as mutual gaze. Either way, it is possible that there may be some discrepancies in results between manually coded and eye tracked studies.

This review has identified a variety of methodological approaches that are likely to affect eye gaze behavior in communication. The review is unable to provide a set of specific design guidelines for future studies on eye gaze behavior in communication, as these would very much depend on the research question and resources available. However, the authors would like to highlight the importance of clearly defining the study variables, such as eye gaze, speech turns or action sequences, to allow for easier comparison. The authors recommend defining gaze in terms of minimum gaze fixation in milliseconds (e.g., 66 ms—Eberhard and Nicholson, 2010, 120 ms—Zima et al., 2019, 200 ms—Ijuin et al., 2018) and explaining the reasons for chosen fixation duration. When choosing ways to define speech, the authors recommend applying a more developed approaches such as CA (Schegloff, 1972; Schegloff and Sacks, 1973; Sacks et al., 1974; Stivers and Enfield, 2010) or MUMIN (Allwood et al., 2007). Adopting these definitions would provide consistency in future research. As observed in this review, failing to report the key information in qualitative studies is a common phenomenon that makes evaluation of the literature difficult (O'Brien et al., 2014). The authors would recommend that qualitative studies provide more details about their methodological approach as often it was unclear what population or in what situations the results could be applied. Future studies could use this review as a guide on what key variables should be considered in the design stage and reported in publications.

As for this review itself, it is important to note, that although the formation of the research question and defining of research terms were carried out by both authors, the searching and data extraction were completed by the first author which may have introduced unintentional bias. This was addressed by checking and validating the emerging themes through discussion of the evidence from the reviewed studies. The readers should be aware that the review was done by searching only two databases and has only included literature written in the English language. As such this may have implications for the reported results, as other important publications may have been missed out, as demonstrated by additional papers identified during the reference scan. Furthermore, the review excluded papers that explored gaze behavior in a clinical population, which likely missed results reported from healthy control groups (as these were excluded by the search terms). However, it is hoped that the findings can be of use for researchers working with clinical and non-clinical populations in developing their research questions and methodologies. The review is also limited to a healthy adult population, so it is unclear if the same eye gaze patterns would follow with children, teenagers or people with mental health conditions.

## Conclusions

In conclusion, there is a clear evidence that eye gaze plays a role in communication, whether that is in each turn during speech or in relation to exchange sequences that take multiple turns to complete. More specifically, there is strong support that eye gaze facilitates turn yielding, plays a role in speech monitoring, prevents and repairs conversation breakdowns and facilitates intentional and unintentional speech interruptions. However, when it comes to starting a turn, the results are somewhat more variable with several modifiers that influence gaze behavior. Kendon (1978) argued that the difference between his (1967) study and studies carried out by Rutter et al. (1978) and Beattie (1978), was a product of different study designs. The studies summarized here used a wide range of methodologies, frequently failing to present what motivated their design decision-making, yet the majority reported similar study outcomes. Whilst there is a lot of evidence to suggest eye gaze plays a role in regulating conversations, it must not be forgotten that other signals such, as intonation and gestures (Duncan, 1972) may also help to inform the next speaker of their turn. Jones and LeBaron (2002) pointed out that much of the research on communication concentrates on verbal and non-verbal behaviors separately and suggested these should be studied as related phenomena. Future studies should learn from the work conducted over the past 50 years to avoid (i) repetition and (ii) guide their methodological decision-making. Particularly important is to agree definitions of key variables (i.e., eye gaze, turn) for easy comparison and all methodological decisions (e.g., dyad or triad, conversation topic, etc.) should be justified. This review provides a good starting point for future studies to understand the basics of eye gaze in turn taking, make informed decisions about study methods for examining eye gaze and selecting variables of interest.

## Author Contributions

ZD and AA formulated the research question, defined research terms, validated the emerging themes through discussion of the evidence from the reviewed studies, and agreed on the final version of the manuscript. ZD completed the searching and data extraction, wrote the initial manuscript, and prepared tables. AA provided critical feedback and made revisions to the manuscript. All authors contributed to the article and approved the submitted version.

## Conflict of Interest

ZD and AA were employed by Samsung AI Center, Cambridge, at the time of this research.
